# New Possibilities in the Treatment of Brief Episodes of Highly Symptomatic Atrial Tachycardia: The Usefulness of Single-Position Single-Beat Charge Density Mapping

**DOI:** 10.1161/CIRCEP.121.010340

**Published:** 2021-10-26

**Authors:** Rita B. Gagyi, Anna M.E. Noten, Krista Lesina, Bakhtawar K. Mahmoodi, Sing-Chien Yap, Mark G. Hoogendijk, Sip Wijchers, Rohit E. Bhagwandien, Tamas Szili-Torok

**Affiliations:** Department of Cardiology, Electrophysiology, Erasmus MC, University Medical Center Rotterdam, the Netherlands.

**Keywords:** cardiac imaging techniques, catheter ablation, humans, recurrence, tachycardia

Brief episodes of atrial tachycardias (ATs) are difficult to locate and ablate using sequential electrophysiology mapping techniques.^1^ In the majority of cases, these patients are either not accepted for ablation, the attempt is unsuccessful, or they develop frequent recurrences after catheter ablation.^2^ Pace mapping is an alternative for a subset of patients with short-lived ATs; however, mapping various mechanisms of AT is challenging for this technique as well.^3^ A novel mapping modality provides the possibility of global mapping, which hypothetically eliminates most of the current limitations.^4^ The AcQMap system (Acutus Medical, Carlsbad, CA) allows for noncontact mapping of a single atrial beat. We aimed to demonstrate the value of this novel mapping technique in the treatment of short-lived ATs.

The data that support the findings of this study are available from the corresponding author upon reasonable request. In this retrospective study, we investigated outcomes of catheter ablation procedures in patients with brief episodes of AT using a novel mapping modality (AcQMap). We defined brief episodes of AT as previously unmappable cardiac arrhythmia, using standard sequential mapping techniques. We selected a cutoff value of maximum 5 minutes AT duration as inclusion criteria, which assumed that sequential mapping was unfeasible. We screened patients with supraventricular tachycardias referred for catheter ablation utilizing the AcQMap mapping system. After an initial exclusion of patients with atrial fibrillation and sustained mappable AT, we included 2 groups of patients with brief episodes of AT. A group of patients who were referred for redo procedure due to frequent recurrences after the previous procedure(s), and a group of de novo patients, which consisted of patients who were initially planned with the AcQMap technology, and patients who were previously rejected from catheter ablation because AT episodes were considered unmappable. Baseline demographic and clinical characteristics from patients were collected in accordance with the hospital institutional review board policies. Procedural data were derived from the electronic medical files and procedural log files recorded by the AcQMap system. This study was approved by the institutional review committee (the ACUTUS registry MEC-2018-1640); all subjects gave informed consent.

AcQMap is a high-resolution imaging and mapping system that uses ultrasound to reconstruct real-time endocardial anatomy and an inverse algorithm to derive endocardial charge density. This technology has been described elsewhere.^4^ After reconstructing the anatomy and overlaying high-resolution charge density maps of electrical activation, ablation was performed using either Celsius ThermoCool (Biosense Webster) or MagnoFlush (Medfact) ablation catheters (Figure [A]). Using this system in combination with short-lived arrhythmias, all ablation points are done exclusively based on the AcQMap making the operator blinded to electrogram data. Following the ablation procedure, follow-up visits were planned for every patient based on institutional methodology plan.

**Figure.**
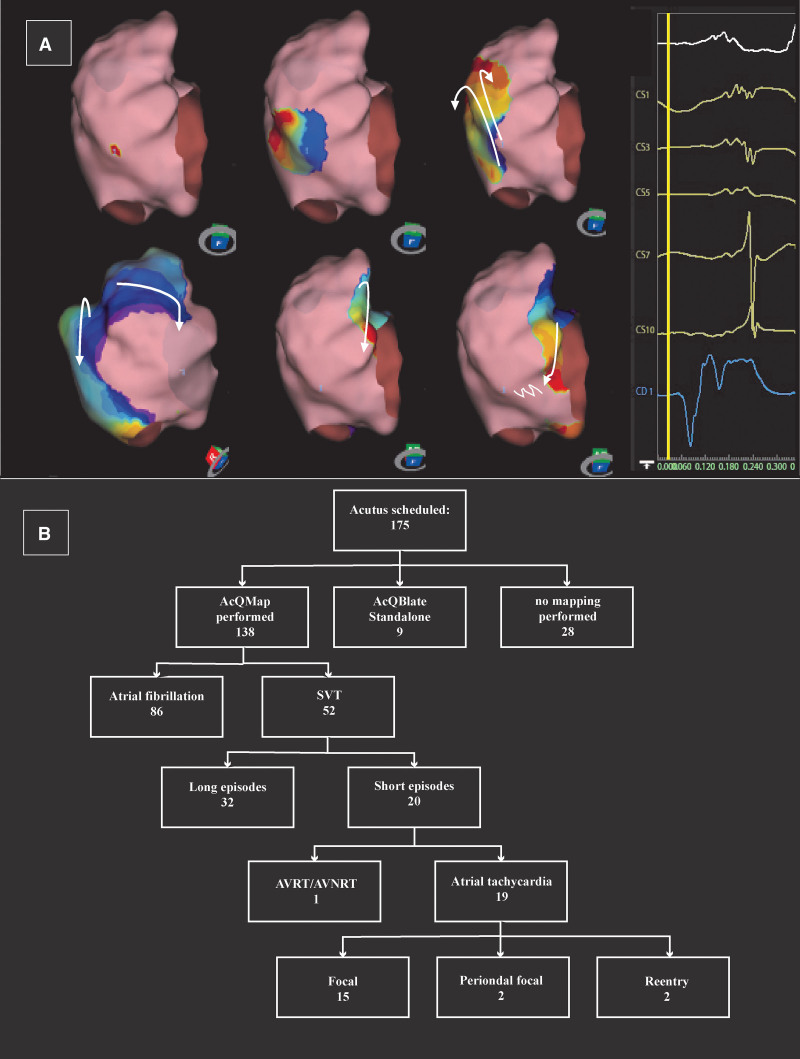
**Figure presenting a propagation map, and patient selection. A**, Example of a patient presenting atypical flutter tachycardia originating in the right atrium (RA). A modified inferior view of the RA representing the propagation history maps with critical isthmus on the medial posterolateral wall. The color red is used to indicate the leading edge of the wavefront with the trailing color bands showing earlier locations of the wavefront. CD1 represents the local charge density signal at the breakout point in the critical isthmus with a QS morphology. CS activation and lead I are also represented. **B**, A total number of 175 patients were referred for catheter ablation with scheduled Acutus mapping method. AcQMap was performed in 138 cases. Twenty patients with short episodes of atrial tachycardia were identified. AVNRT indicates atrioventricular nodal reentry tachycardia; AVRT, atrioventricular reentrant tachycardia; CD1, local unipolar charge signal; CS, coronary sinus; and SVT, supraventricular tachycardia.

To compare data between patient groups, we used independent *t* test and Mann-Whitney *U* test in SPSS software. Primary end point was procedural efficacy, and secondary end point was long-term outcome defined as the number of recurrences.

Twenty of 175 patients (men, n=4; women, n=16) had brief episodes of AT (Figure [B]). Fourteen underwent a repeat procedure (redo group); 6 patients had a de novo procedure (de novo group). The average time between onset symptoms and the final procedure was 46±49 months (mean±SD) and was significantly shorter in the de novo than redo group (15±14 versus 59±54; *P*=0.02). Total procedural time was 160±46 minutes, with a total fluoroscopy time of 16±8 minutes. Total radiofrequency application duration was 653±444 s. Left atrial localization of AT was identified in 50% of patients, right atrium localization in 37.5%, and septal origins in 12.5% of patients. Regarding the mechanism of the mapped arrhythmias, we found atrioventricular reentrant tachycardia/atrioventricular nodal reentry tachycardia in 1 patient, focal AT in 15, perinodal focal in 2, and reentry mechanism in 2 patients (Figure [B]). An average number of 1.1 of high-resolution charge density maps were performed in the left atrium and 2.0 in right atrium. In all cases, focal activation pattern was identified on the AcQMaps as target of ablation. Acute success was achieved in 19 of 20 (95%). In 1 patient, ablation was unsuccessful because of parahisian location of a perinodal reentry circuit. Recurrence during follow-up developed in one additional patient (5%). Using AcQMap, we documented mapping times of 3.2±2.5 minutes.

The current study found that brief episodes of highly symptomatic AT can be mapped using single-position single-beat charge density mapping (AcQMap) and ablated successfully with high acute and long-term success rate. In addition, AT can be eliminated in a shorter period of time, when patients are scheduled directly for AcQMap-guided procedures. When comparing our methods and results to previous studies, it must be pointed out that all studies included only patients with sustained, long episodes of AT, and patients with short-lasting arrhythmias were not even considered for treatment. Therefore, our results can be interpreted as the first-in-human systematic report of an interventional approach for patients experiencing brief episodes of AT.

## Article Information

### Sources of Funding

None.

### Disclosures

None.
